# Early characterization and prediction of glioblastoma and brain metastasis treatment efficacy using medical imaging-based radiomics and artificial intelligence algorithms

**DOI:** 10.3389/fonc.2025.1497195

**Published:** 2025-01-30

**Authors:** Noémie N. Moreau, Samuel Valable, Cyril Jaudet, Loïse Dessoude, Leleu Thomas, Romain Hérault, Romain Modzelewski, Dinu Stefan, Juliette Thariat, Alexis Lechervy, Aurélien Corroyer-Dulmont

**Affiliations:** ^1^ Medical Physics Department, Centre François Baclesse, Caen, France; ^2^ Université de Caen Normandie, CNRS, Normandie Université, ISTCT UMR6030, GIP CYCERON, Caen, France; ^3^ Radiation Oncology Department, Centre François Baclesse, Caen, France; ^4^ UMR GREYC, Normandie Univ, UNICAEN, ENSICAEN, CNRS, Caen, France; ^5^ LITIS - EA4108-Quantif, University of Rouen, Rouen, France; ^6^ Nuclear Medicine Department, Henri Becquerel Center, Rouen, France; ^7^ ENSICAEN, CNRS/IN2P3, LPC UMR6534, Caen, France

**Keywords:** Glioblastoma (GBM), machine learning (ML), brain tumors, artificial intelligence, treatment efficacy, medical imaging, radiotherapy

## Abstract

Among brain tumors, glioblastoma (GBM) is the most common and the most aggressive type, and brain metastases (BMs) occur in 20%–40% of cancer patients. Even with intensive treatment involving radiotherapy and surgery, which frequently leads to cognitive decline due to doses on healthy brain tissue, the median survival is 15 months for GBM and about 6 to 9 months for BM. Despite these treatments, GBM patients respond heterogeneously as do patients with BM. Following standard of care, some patients will respond and have an overall survival of more than 30 months and others will not respond and will die within a few months. Differentiating non-responders from responders as early as possible in order to tailor treatment in a personalized medicine fashion to optimize tumor control and preserve healthy brain tissue is the most pressing unmet therapeutic challenge. Innovative computer solutions recently emerged and could provide help to this challenge. This review will focus on 52 published research studies between 2013 and 2024 on (1) the early characterization of treatment efficacy with biomarker imaging and radiomic-based solutions, (2) predictive solutions with radiomic and artificial intelligence-based solutions, (3) interest in other biomarkers, and (4) the importance of the prediction of new treatment modalities’ efficacy.

## Introduction

Brain tumors are highly heterogeneous neoplasms not only from a histological point of view but also from an intratumor temporal and spatial perspective.

Despite treatments including surgery, chemotherapy and radiotherapy patients with brain tumors respond heterogeneously. The same treatment will result in different treatment outcomes. Treatment efficacy is currently evaluated using anatomical MRI several months after treatment initiation. Differentiating non-responders from responders as early as possible in order to tailor treatment in a personalized medicine fashion to optimize tumor control is the most pressing unmet therapeutic challenge.

In this review, we will provide an overview of current research on treatment response assessment for a very aggressive and brain tumor called glioblastoma (GBM) and for a frequent brain tumor: brain metastasis (BM). To provide a clear structure and taxonomy of the reviewed literature, we have categorized the studies into the following sections:

Introduction:

- Overview of brain cancer and the therapeutic challenge of early characterization and prediction of treatment response.

Early characterization of brain cancer treatment efficacy:

- Review of studies using functional imaging biomarkers with MRI, PET, and CT with intensity thresholding for early detection in the days after treatment initiation.

Prediction of treatment response:

- Brief introduction to radiomics and its potential in medical imaging and treatment response assessment.
- Studies utilizing radiomics for extracting quantitative features from clinical routine MRI as input for predicting treatment response in brain cancer patients before its initiation.
- Brief introduction to AI and its potential in medical imaging and treatment response assessment.
- Research on various machine learning (ML) algorithms [e.g., support vector machines (SVMs), random forests, and neural networks] and studies using deep learning (DL) techniques, such as convolutional neural networks (CNNs), recurrent neural networks (RNNs), and transformers to predict treatment outcome before its initiation.

Challenges and future directions for assessment of new treatment efficacy.

**Figure 1:**
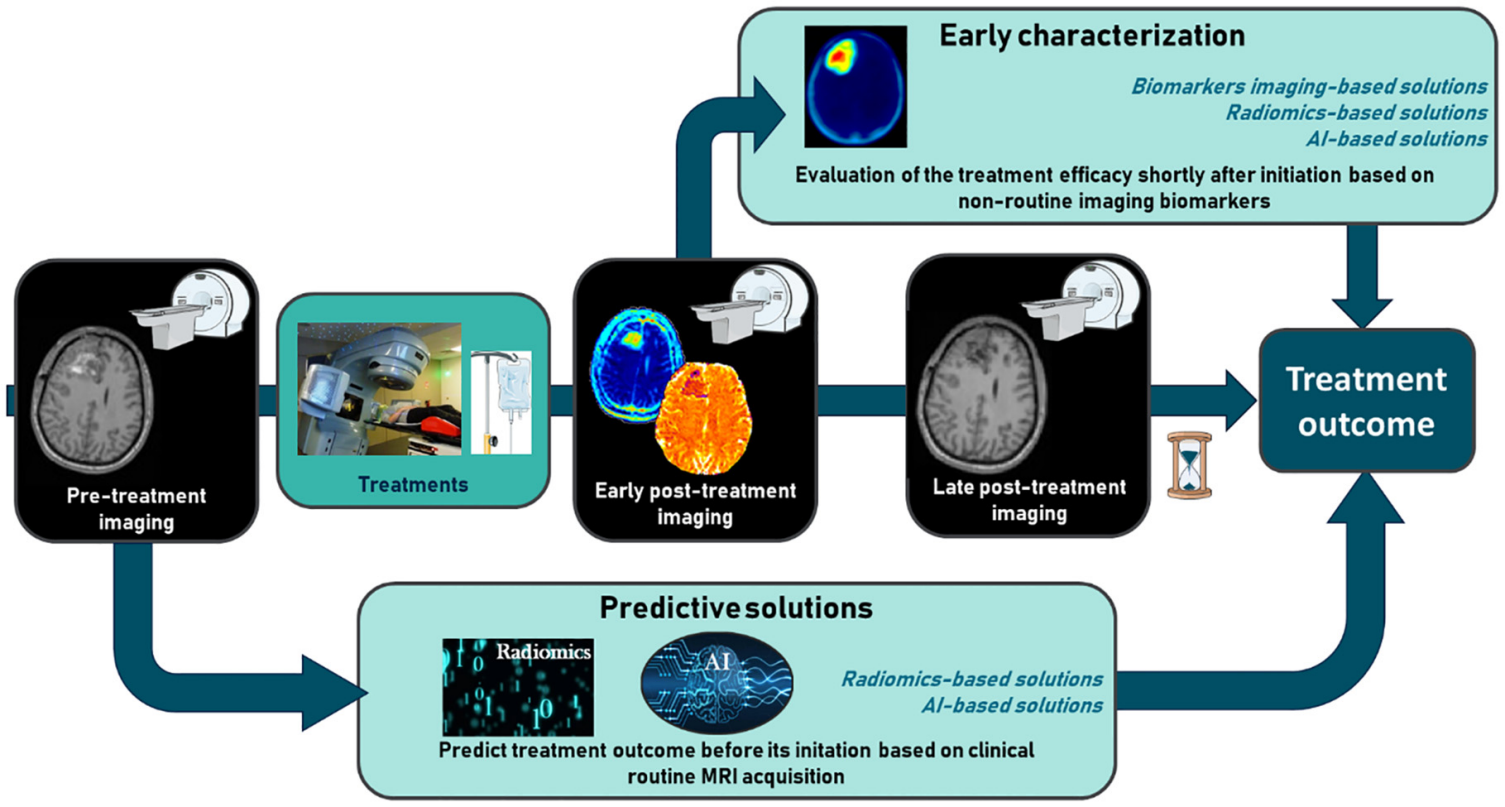
The challenge of early characterization in predicting therapeutic efficacy in glioblastoma and brain metastases.

Following the structure of the research and the taxonomy above, in this review, we will firstly focus on early characterization, which involves evaluation shortly after treatment initiation and mainly relies on imaging biomarkers/readouts. We will then focus on the ability to predict treatment efficacy before its initiation using radiomics and new innovative approaches using artificial intelligence (AI) (**Figure 1**). AI aims to mimic human intelligence through algorithms executed in a computer environment. AI algorithms are increasingly being studied in the field of medical imaging, whether for image processing, diagnosis, or the prediction of patient prognosis (1). One of the benefits of AI is its ability to handle large datasets and extract relevant information that is difficult to obtain through human intelligence. For those reasons, more important focus was made on AI solutions.

### Article selection methodology

Databases: We conducted a comprehensive search using multiple databases, including PubMed, Web of Science, and Google Scholar. The search terms used were “Artificial Intelligence,” “radiomics,” “brain cancer,” “treatment response,” “glioblastoma,” “brain metastases,” “prediction,” “machine learning,” “deep learning,” and related synonyms.

Time frame: We considered articles published from January 2012 to the present to ensure the inclusion of the most recent advancements.

Selection process: Articles were selected based on their novelty, number of patients, unique or multicenter approaches, and relevance to the therapeutic challenge.

Study design: We included original research articles and review papers; no case studies were included.

Quality: Only peer-reviewed articles from reputable journals and conferences were considered to ensure the reliability and validity of the findings.

Data extraction: Relevant data, including study design, novelty of the AI techniques used, outcomes, and limitations, were extracted from each selected article.

### Current management of brain tumors

GBM is the most common and most aggressive primary brain tumor. Despite treatments including surgical resection, radiotherapy, and chemotherapy, the overall survival remains low (survival median of 15 months) with a high rate of tumor recurrence (2). While GBM has an incidence of 3.22 per 100,000 (3), BMs affect 20% to 40% of cancer patients (4) and represent the most common primary tumor with an incidence 3 to 10 times higher than primary brain tumors (5). BMs occur more frequently in patients with melanoma, lung cancer, or breast cancer (70%, 40%, and 20%, respectively (6)). As for GBM, despite aggressive treatment with radiotherapy and surgery that often led to cognitive decline due to healthy brain tissue dose toxicity, the survival median for patients with BM is very short and is about 6 to 9 months from the diagnosis of BM (7).

### Therapeutic challenges

Patients with GBM (as well as patients with BM) present heterogeneous treatment responses (8). For the standard treatment (corresponding to surgery plus Stupp regimen), some GBM patients (a minority) are responders and present overall survival higher than 30 months and others are non-responders and die in few months (9). The pressing unmet therapeutic need is to be able to discriminate as soon as possible the non-responder patients from the responders to adapt treatment in a personalized medicine manner to optimize tumor control as well as healthy brain tissue preservation.

The process of evaluating therapeutic response is similar for GBM and BM. The assessment is mainly based on response evaluation criteria in solid tumors (RECIST) (10) and response assessment in neuro-oncology (RANO-BM) (11) criteria, which evaluate the evolution of lesion size on anatomical MRI, at different times after the treatment.

However, the issue is that assessment of the efficacy or non-efficacy of therapies, using conventional anatomical MRI, is only possible approximately 2 months after the beginning of treatment (12). Indeed, there is too much pseudoprogession or inflammatory response before and anatomical MRI is only able to reach the morphological aspect of the tumor. Focusing on other imaging biomarkers that are more specific to tumor biology could help shorten this wasted time, allowing for earlier assessment of treatment efficacy (13).

## Subsections relevant for the subject

### Early characterization of treatment efficacy

#### Biomarker imaging-based solutions

As shown in **Table 1A**, several publications have explored which imaging biomarkers might be more effective than anatomical MRI in predicting early therapeutic response (chemotherapy combined with anti-angiogenic therapy) and overall survival in patients with GBM and recurrent GBM at the clinical and preclinical level. Li and colleagues (14) have shown, on patients, that [^18^F]-AlF-NOTA-PRGD2 PET/CT ([^18^F]-RGD PET/CT) and dynamic contrast-enhanced MRI (DCE-MRI) can assess response to treatment, demonstrating that a greater decrease in SUV mean predicts better progression-free survival. Magnetic resonance spectroscopy (MRS) can predict early treatment efficacy. Talati et al. (15) performed a longitudinal MRI/MRS to study whether changes in N-acetylaspartate (NAA)/Choline (Cho) and Lactate (Lac)/NAA from different times after treatment can predict early therapy failures. Changes noted in metabolic levels of NAA/Cho and Lac/NAA were able to predict treatment failure as early as 1 day after anti-angiogenic treatment. This is in accordance with the review made by Qi and colleagues (16), who showed the different modalities and biomarkers that enable early characterization of therapeutic efficacy. At the preclinical level, Corroyer-Dulmont et al. have shown that [^18^F]-fluoro-thymidine ([^18^F]-FLT PET) (marker of cell proliferation), compared with other PET {[^18^F]-fluorodeoxyglucose ([^18^F]-FDG PET)} or MRI biomarkers, can characterize treatment efficacy from 3 days after treatment initiation, at a time when anatomical MRI shows no differences (17). Predicting treatment efficacy in recurrent GBM is also an important therapeutic challenge. One clinical study and one preclinical study have shown the importance of using [^18^F]-FLT PET to predict progression-free survival and overall survival in recurrent GBM (18, 19).

**Table 1:** Biomarker imaging-based solutions for the early characterization of treatment efficacy (A), radiomic-based solutions for treatment efficacy prediction (B), and AI-based solutions for treatment efficacy prediction (C).

Table 1A Biomarker imaging-based solutions for the early characterization of treatment efficacy.								
Studies	Cohorts (*n*)	Tumor type	Treatment	Imaging modality	Imaging schedule	Outcome prediction	Results	Reference
Clinical	20 patients	GBM	Anti-angiogenic (Bevacizumab) plus conventional radiotherapy and chemotherapy (Temozolomide) (CRT) Adjuvant chemotherapy (Temozolomide) plus anti-angiogenic (Bevacizumab)	^18^F-RGD PET/CT DCE-MRI	Before CRT Before anti-angiogenic Seven weeks after anti-angiogenic	Treatment efficacy	Prediction of response to treatment after 3 weeks	(14)
Clinical	33 patients	Recurrent GBM	Anti-angiogenic (Bevacizumab) monotherapy or combination therapy	MRI/MRS (NAA/Cho and Lac/NAA)	1 day and 2, 4, 8, and 16 weeks after treatment	Treatment efficacy	Prediction of treatment failure to therapy 1 day after treatment	(15)
Preclinical	25 rats and 29 rats	GBM (U87 and U251: human cell line)	Chemotherapy (Temozolomide), anti-angiogenic (Bevacizumab), or both	Anatomical MRI Diffusion MRI CBV MRI [^18^F]-[FLT] PET [^18^F]-FDG PET	5, 10, or 12 days after treatment	Treatment efficacy	[^18^F]-FLT was more predictive: 3 days after initiation treatment	(17)
Preclinical	49 rats	Recurrent GBM (Human U251 cell line)	Chemotherapy (Temozolomide), anti-angiogenic (Bevacizumab), or both	Anatomical MRI Diffusion MRI CBV MRI [^18^F]-[FLT] PET [^18^F]-FDG PET	3, 10, and 17 days after treatment	Treatment efficacy	[^18^F]-FLT was more predictive: 3 days after the end of treatment	(18)
Clinical	30 patients	Recurrent malignant glioma	Chemotherapy (Temozolomide) and anti-angiogenic (Bevacizumab)	Anatomical MRI [^18^F]-FLT PET	MRI: 6 weeks after treatments PET: 1 to 5 days and at 2 and 6 weeks after treatments	Treatment efficacy	[^18^F]-FLT can be used to determine the treatment efficacy 2 weeks after treatments	(19)

CBV, cerebral blood volume; Cho, choline; CRT, radiotherapy and temozolomide; CT, computed tomography; DCE, dynamic contrast-enhanced; [^18^F]-FDG, [^18^F]-fluorodeoxyglucose; [^18^F]-FLT, [^18^F]-fluoro-thymidine; GBM, glioblastoma; Lac, lactate; MRI, magnetic resonance imaging; MRS, magnetic resonance spectroscopy; NAA, N-acetylaspartate; PET, positron emission tomography; [^18^F]-RGD, [^18^F]-AlF-NOTA-PRGD2.

**Table 1B:** Radiomic-based solutions for treatment efficacy prediction.

Studies	Cohorts (*n*)	Tumor type	Treatment(s)	Imaging modality	Features numbers	Models	Outcome prediction	Results	Reference
Clinical	237 patients	BM	Gamma knife radiosurgery (GKRS)	MRI	Clinical: 5 Radiomic: 4	SVM	Overall survival	Radiomics and clinical features combination (AUC = 0.82, Acc = 0.80, Sens = 0.77, Spe = 0.81)	(24)
Clinical	256 patients	BM	GKRS	MRI	Clinical: 5 Radiomics: 5	SVM	Local tumor control	Radiomics and clinical features combination (AUC = 0.95, Acc = 0.89, Sens = 0.87, Spe = 0.91)	(24)
Clinical	125 patients	GBM	Radiotherapy and concomitant chemotherapy (Temozolomide)	MRI	Clinical: 6 Radiomics: 21	RF, SVM, LR	Survival stratification	Radiomics and clinical features combination (AUC = 0.92)	(25)
Clinical	76 patients	GBM	Chemoradiotherapy	MRI	Clinical: 2 Radiomics: 6	Naïve Bayes	Distinction in early true progression between pseudoprogression	Radiomics and clinical features combination (AUC = 0.80, Acc = 0.737, Sens = 0.78, Spe = 0.67)	(26)
Clinical	337 patients	BM	SRS	MRI	Clinical: 4 Radiomics: 223	GNB, kNN, RF, AB, SVM, MLP	Treatment response	Best classifier: SVM Radiomics and clinical features combination (AUC = 0.95)	(27)
Clinical	87 patients	BM	Stereotactic radiosurgery (SRS)	MRI	Clinical: 3 Radiomics: 9	RF	Local tumor control	Radiomics and clinical features combination (AUC = 0.79)	(28)

AB, adaptive boosting; Acc, accuracy; AUC, area under the ROC curve; BM, brain metastasis; GBM, glioblastoma; GKRS, gamma knife radiosurgery; GNB, Gaussian naïve Bayesian; kNN, k-nearest neighbors; LR, logistic regression; MLP, multilayer perceptron; MRI, magnetic resonance imaging; RF, random forest; Sens, sensitivity; Spe, specificity; SRS, stereotactic radiosurgery; SVM, support vector machine.

**Table 1C:** AI-based solutions for treatment efficacy prediction.

Studies	Cohorts (*n*)	Tumor localization	Treatment	Imaging modality	Models	Outcome prediction	Results	Reference
Clinical	124 patients	BM	Stereotactic radiation therapy (SRT)	MRI	MLP/Clinical features CNN + Seq2Seq/Transformers/LSTM CNN + Seq2Seq/Transformers/LSTM + clinical features	Local tumor control	CNN + LSTM + clinical features (AUC = 0.86, Acc = 0.83, Sens = 0.77, Spe = 0.87)	(36)
Clinical	30 patients	Gliomas (15 GBM)	/	MRI	HD-GLIO-XNAT (https://github.com/NeuroAI-HD/HD-GLIO-XNAT)	Evaluate whether AI-assisted decision support provides a more reproducible and standardized assessment of response to treatment compared to manual measurements using RANO criteria	Lower-grade gliomas (CCP = 0.77 for RANO and 0.91 with AI)	(37)
Clinical	133 patients	GBM	/	MRI	ANN with clinical features	Survival classification	Cross validation: Acc = 0.91	(38)
Preclinical	28 mice	GL261	Chemotherapy (Temozolomide)	MRI/MRS	1D-CNN, LR, SVM, RF, XGBoost	Therapy response assessment	1D-CNN (Acc = 0.9975, Sens = 0.99, Spe = 0.99)	(39)

Acc, accuracy; AI, artificial intelligence; AUC, area under the ROC curve; BM, brain metastasis; CCP, concordance correlation coefficients; CNN, convolutional neural network; GBM, glioblastoma; LR, logistic regression; LSTM, long short-term memory; MLP, multilayer perceptron; MRI, magnetic resonance imaging; MRS, magnetic resonance spectroscopy; RANO, response assessment in neuro-oncology; RF, random forest; Sens, sensitivity; Spe, specificity; SRS, stereotactic radiosurgery; SVM, support vector machine; XGBoost, extreme gradient boosting.

However, early characterization has a limitation. Even if it is effective, the patient has already undergone treatments (radiotherapy and chemotherapy) and may be exposed to their side effects (20).

The recent development of innovative computer techniques such as radiomics or more recently AI could lead to predict treatment effectiveness before its initiation. This will lead to a more personalized medicine where non-responder patients will gain precious months without undergoing an unnecessary costly treatment that could potentially lead to adverse effects (21).

### Predictive solutions

#### Radiomic-based solutions

The term “radiomics” first appeared in literature in 2012 through an article published by Lambin et al. (22). This approach, focused on medical imaging data, aims to extract a large set of features from an image for a better characterization of tumor. Radiomic protocols require the following six steps: image acquisition, image reconstruction and pre-processing, segmentation, resampling, features extraction, and features selection and model-based feature construction (23). Because of these various steps, the use of radiomics aims to be potentially predictive compared to imaging biomarker analyses based on basic features such as mean or peak intensity. Image characteristics are subjected to a more in-depth analysis, making the features more relevant for prediction, and consequently, the results are more effective. Radiomics models are capable of predicting therapeutic response or overall survival (23).

In that context, the use of radiomics to develop models capable of predicting treatment response prior to brain tumor treatment initiation has been explored in several studies.

One of these studies (24) investigated the extraction of radiomic features from post-treatment MRI in patients with BM to predict local tumor control with an estimation of the tumor volume percentage compared to pre-treatment and overall survival with 256 and 237 patients, respectively. Three models were constructed through the training of SVMs using a Gaussian kernel and Bayesian optimization for hyperparameter tuning: (i) clinical features (age, gender overall survival, numbers of tumors, local tumor control, and median dose), (ii) radiomic features, and (iii) combined clinical and radiomic features. For both prediction objectives, the model combining clinical and radiomic features achieved very interesting performances with an area under the receiver operating characteristic curve (AUC) of 0.95 for local tumor control and 0.82 for overall survival.

Furthermore, a clinical study (25) was conducted to predict survival stratification of 125 patients with GBM. Radiomic features were extracted from MRI images. Among the three tested ML models, the SVM model demonstrated the best performance, with an AUC of 0.92.

**Table 1B** (24–28) summarizes several studies on the prediction of treatment response based on radiomics obtained from pre-treatment imaging. In all studies, the AUC is between 0.62 and 0.95. All these studies highlighting combining radiomic features with clinical features enhance prediction performance. However, radiomics has some limitations for routine clinical application. Most published studies have a relatively small patient cohort especially for GBM. However, to develop effective models, a sufficiently large training and test set is mandatory (29). Because of its complexity, radiomics presents the challenge of low interpretability of the features and models used, raising caution among physicians regarding the use of radiomics models in clinical settings (30). Beyond these points, the main limitation of radiomics remains the low stability and inter-hospital portability of the models (29). To resolve to this challenge, initiatives like the “Imaging Biomarker Standardization Initiative (IBSI)” (31) have been developed to complement radiomic features extraction; however, the robustness of these predictive models remains an issue before their adoption as a standard of care as shown by Peerlings and colleagues (32) for diffusion MRI or CT (33) or even for Test–Retest in PET imaging (34).

Therefore, it is timely to explore more innovative current developments in AI that may enable predictive characterization of treatment efficacy. DL is known to be able to extract more complex and a larger number of features in medical imaging than radiomics, which could lead to better performance (35).

#### Artificial intelligence-based solutions

Several studies have evaluated the use of AI algorithms to assess the therapeutic efficacy of GBM and BM. A clinical study (36) involving 124 patients with BM developed a CNN-based architecture to extract features from each MRI slice to predict the outcome of local control/failure in BM treated with stereotactic radiation therapy. A CNN is a type of DL neural network specifically designed to process structured data arrays, such as images. They integrated an InceptionResentV2 CNN architecture and a transformer (to consider spatial dependences between MRI slices during modeling). Depending on the mechanism of integration of information from each MRI slice, the AUC ranged from 0.72 to 0.86. The best performance was obtained with the combination of DL features obtained from anatomical MRI with clinical variables (tumor size, age, gender, tumor location, histology, total dose, previous WBRT, and number of BM).

In a study including 30 patients (15 with low-grade glioma and 15 with GBM), Vollmuth et al. (37) demonstrated that AI using artificial neural network (ANN) for brain and then tumor segmentations has the potential to provide a more reproducible and standardized assessment of treatment response on MRI compared to manual two-dimensional measurements of tumor burden using RANO criteria. Time to progression (TTP) was initially evaluated according to RANO criteria based on MRI and then revaluated by incorporating additional information from AI-enhanced MRI sequences that describe longitudinal changes in tumor volume. The inter-observer concordance correlation coefficient (CCC) for TTP measurements was 0.77 using the RANO criteria alone. With the addition of AI, the CCC increased to 0.91. This improvement was most observed in patients with low-grade gliomas (0.70 without AI vs. 0.90 with AI). Because of the less aggressive nature of these tumors, reliable assessment of TTP can be more difficult.

In a previous study, Luckett et al. (38) show a good performance with an accuracy of 90.6% in classifying survival (<1 year, 1–2 years, and >2 years), employing a deep feedforward CNN comprising three hidden layers with eight neurons in each layer to predict patient survival in a cohort of 133 individuals. Ortega-Martorell and colleagues also showed a good performance of one-dimension CNN in a preclinical study to track therapy response in GBM (39). The 1D-CNN performed better than different ML models, showing the superiority of DL methods.

Our review of the literature reveals that the CNN exhibits superior performance. Although the architecture is not novel, it is particularly suited to medical imaging and currently offers the most effective means of predicting treatment efficacy (40).

**Table 1C** (36–39) summarizes several studies on the prediction of treatment response based on AI algorithms from pre-treatment MRI. As in the radiomics-based studies, the best performance is achieved by combining imaging data with clinical information.

Many studies applying AI in this field are based on relatively small data cohorts (less than 100 for GBM). However, a large data cohort is essential for optimal training of AI models (41). Centralizing a large amount of data in a single center can be challenging, and the performances of models are not always transferable between centers. Federated learning (42) addresses this issue by enabling learning from distributed data without transferring it between sites. Federated learning is a DL paradigm in which a model is trained across multiple decentralized devices or servers located in various medical centers, each holding local data samples, without the need to exchange the raw data. The only parameters shared among the different hospitals are the model parameters, not the raw medical data.

In addition, AI methodology is constantly evolving and new architectures appear every year. The models we have presented in this review give an overview of what is being done today, but new architectures such as diffusion models or full transformers should be more present in the years to come. One example is the UNEt TRansformer (UNETR) (43), which adapts the CNN encoder/decoder models proposed by UNET to transformer architectures in order to process sequential representations of the input volume more efficiently. Transformers are a type of AI model designed to efficiently process sequential data, such as text. Functional imaging such as proliferation index or other indicators is more relevant for assessing therapeutic efficacy (17). To our knowledge, no study involving AI models uses functional imaging biomarkers for predicting GBM efficacy as all the articles reported in this review used clinical routine anatomical MRI. However, in other cancers with radiomic models, Knuth and colleagues as well as Zhang and colleagues support the add value of function biomarkers in comparison to anatomical MRI in rectal (44) and breast cancers, respectively (45).

Opting for more functional imaging biomarkers instead of anatomical MRI could potentially improve AI performance in predicting treatment efficacy.

It is important to note that current studies were based on 2016 WHO classification rather than the 2021 one. To the best of our knowledge, no study has yet evaluated the potential of AI models to predict treatment outcomes of GBM according to the WHO 2021 classification. These models may not fully reflect current standards and advancements in the field, potentially leading to biases in predictions. However, current performances of the AI models to predict treatment outcome are still valid if they do not take into account the grade of the tumor, for example, if the input data only take the pre-treatment MRI. If the model is capable of predicting the treatment outcome of a brain lesion on an MRI, it should still be able to do so regardless of whether the brain lesion is designated as a GBM or a grade 4 astrocytoma Therefore, it is essential to incorporate recent classifications to ensure that AI models are aligned with best clinical practices and provide reliable and relevant recommendations.

### AI models to distinguish pseudo-progression to recurrence

For patients with GBM treated in accordance with the established standard protocol, the prevalence of pseudoprogression is estimated to range between 20% and 30%. This phenomenon typically manifests within 1 to 12 weeks following the conclusion of treatment and is distinguished by an increase in tumor volume and the emergence of new lesions discernible on magnetic resonance imaging (MRI) (46).

This represents a significant challenge in clinical routine, as it complicates the assessment of treatment response and may impact therapeutic decision-making. Distinguishing between pseudoprogression and tumor recurrence is essential for optimal patient management, but this differentiation requires a significant amount of imaging. The acquisition of earlier information on potential pseudoprogression could enable treatment to be adapted more rapidly. Several studies have shown that radiomics and AI could be pertinent tools to predict pseudoprogression. Sun et al. (47) evaluated the diagnostic performance of ML models using a radiomic model based on contrast-enhanced T1-weighted MRI to differentiate pseudoprogression from true progression after standard treatment for 77 patients. The classifier demonstrated limited results with a sensitivity of 78.36% and a specificity of 61.33%. Another study (48), based on 78 patients with GBM, developed a CNN combined with an LSTM to differentiate anatomical MRI pseudoprogression from progression. The AUC results of the three trained models ranged from 0.52 to 0.83. The model that demonstrated the highest performance was the one that combined both MRI data and clinical features including age at the time of surgery, gender, methylation status of the 06-methylguanine-DNA-methyltransferase (MGMT) promoter, mutational status of the isocitrate dehydrogenase (IDH) gene, the total dose and number of fractions of radiotherapy, and other factors. Moassefi and colleagues (49) developed a DL model to distinguish pseudoprogression from true progression for 124 patients, using only clinical routine MRI. The model achieved a mean accuracy of 76.4%, a mean AUC of 0.76, a mean sensitivity of 88.72%, and a mean specificity of 62.05%.

An article using nuclear medicine imaging shows that radiomics based on FET-PET was able to differentiate tumor progression from pseudoprogression (50). Kebir et al. used FET-PET images in 14 patients and applied an unsupervised clustering algorithm for the diagnosis of pseudoprogression, achieving a diagnostic accuracy of 75%.

These studies demonstrate that it is possible to predict pseudoprogression at a relatively early stage, which could potentially optimize patient management. However, it is important to note that (1) performances of the models are limited with a specificity and a sensitivity of approximately 0.7 to 0.8 and (2) none of these studies have explored the prediction of pseudoprogression using pre-treatment imaging, highlighting a significant area for future research.

### Interest in other biomarkers

This review focuses on the relevance of imaging biomarkers and the use of radiomics and AI based on MRI before and after treatment. However, molecular biomarkers can also be used to characterize therapeutic efficacy and overall survival. One such molecular biomarker is the methylation status of MGMT (51). The 1p/19q codeletion and loss of chromosome 10 are also predictive of therapeutic response (52). Although these biomarkers are used in routine clinical practice, the cost of testing, limited resources, and analysis time may be limiting factors for some patients (53). In contrast, MRI and RT DOSE are performed for each patient.

In addition, a biopsy is only performed on a part of the tumor. Since GBMs are recognized as highly heterogeneous tumors, molecular or protein expression will not be representative of the entire tumor, introducing a variability in the evaluation of therapeutic response (54). Therefore, imaging biomarkers appear to be the most suitable for routine clinical application.

### New treatment modalities

Predicting the efficacy of treatments is of great interest for responder patients. However, for non-responder patients, the use of new treatment modalities, such as proton therapy and carbon ion therapy, is essential. It is important to conduct studies in these areas to assess the appropriateness of using one treatment over another, based on expected therapeutic efficacy. These studies are of crucial importance for the integration of these new treatments, which still need to be validated, especially through clinical trials (55). In this context, an AI tool that predicts treatment efficacy before initiation would be of significant interest.

## Conclusion

The practical applications of AI and radiomics in the management of brain cancer are significant. These technologies enable earlier diagnosis, facilitating rapid and personalized treatment plans. For patients, this translates into better clinical outcomes and improved quality of life, particularly through the rapid identification of cases of non-response to treatment, paving the way to more appropriate therapeutic alternatives. As far as healthcare systems are concerned, AI and radiomics offer the possibility of optimizing the use of resources and reducing the financial impact of costly and ineffective treatments.

However, a number of challenges remain. These include the time and effort required to train healthcare professionals in the use of these technologies, as well as the management of administrative and regulatory obstacles.

The review highlights the pressing need for early and accurate characterization of treatment efficacy in GBM and BMs, given their aggressive nature and the heterogeneous responses to standard treatments. Current methods, relying on anatomical MRI, often fail to provide timely assessments due to pseudoprogression, leading to delayed treatment adjustments and potential cognitive decline from radiotherapy.

### Early characterization of treatment efficacy

Imaging biomarkers, such as PET/CT, DCE-MRI, and MRS, have shown promise in predicting treatment response and overall survival earlier than conventional MRI. However, these methods still require patients to undergo initial treatments, exposing them to potential side effects.

### Predictive solutions

Radiomics and AI offer innovative approaches to predict treatment efficacy before initiation. Studies combining radiomic features with clinical data have achieved high AUC values, indicating strong predictive performance. However, radiomics faces challenges such as low interpretability and limited inter-hospital portability, which initiatives like the IBSI aim to address.

Our review shows that AI, particularly DL techniques like CNNs, has demonstrated superior performance in predicting treatment outcomes. Combining AI-extracted features from MRI with clinical variables has yielded impressive results, with AUC values ranging from 0.72 to 0.99. Federated learning presents a solution to the challenge of data centralization, allowing models to be trained across multiple decentralized sites without exchanging raw data.

### Challenges and future directions

Despite the promising results, several challenges remain. Most studies are based on small patient cohorts, which limits the generalizability of the findings. Additionally, the use of functional imaging biomarkers, which may provide more relevant information than anatomical MRI, has not been extensively explored in AI models for brain efficacy prediction. The integration of radiomics and DL in neuro-oncology has led to significant advancements in the management of gliomas, particularly by exploiting complex imaging features to predict molecular and clinical profiles. However, significant challenges remain, including the harmonization of multimodal data. Future research should focus on developing federated learning frameworks and enhancing model interpretability (56).

### Pseudoprogression and new treatment modalities

Distinguishing pseudoprogression from true progression is crucial for optimal patient management. Radiomics and AI have shown potential in this area; however, the performance of these models is limited, and predicting pseudoprogression using pre-treatment imaging remains an inadequately explored area.

AI and the radiomics model have some limitations that have to be pointed out:

(a) Bias in training data or learning algorithms: Biases in training data represent a major challenge for training AI models. If the dataset used is not representative of the overall population, model performance is likely to degrade, particularly for more diverse patient groups. To limit these biases and better explain model behaviors, a data quality process is essential. This helps to identify and address potential gaps in the distribution of the data used.
(b) AI reliability in a clinical situation, especially with patient populations that are part of more heterogeneous groups: The reliability of AI systems in the clinical setting is a fundamental issue, especially when it comes to treating heterogeneous patient populations. For example, brain tumors such as GBM and BM present great heterogeneity both between tumors and within the same tumor. This diversity can limit the ability of AI models to generalize effectively. To address this, it is essential to rigorously validate these models and continuously adapt them using updated data. In addition, the study of model explainability is essential to understand the decisions made by models.

Moreover, patients included in clinical trials are not representative of the general population of patients in clinical practice because the selection criteria are strict. Consequently, the results of most clinical trials do not allow the same conclusions to be drawn in a different population or context (57).

(c) The challenge of integrating new technology into day-to-day clinical practice: Integrating AI technologies into everyday clinical practice involves a number of challenges. Firstly, sufficiently powerful IT infrastructures are needed to run these models. Secondly, medical staff need to be trained in their use, which can come up against a certain resistance to change. In these cases, the explicability of the models plays a key role in instilling confidence and facilitating their adoption. In addition, it is crucial to develop user-friendly interfaces, integrating these models into practical tools for medical staff. Finally, regulatory and ethical aspects, such as data confidentiality and patient safety, must be considered to ensure the safe and responsible deployment of technologies in the clinical environment.

Articles cited in this review evaluate the performance of the AI models with specificity/sensitivity approaches and not with concrete data from clinical routine experiments or case studies on brain tumor treatment efficacy. A study has developed an AI model for diagnosing breast cancer and determined whether it could be useful to radiologists (58). The study showed that AI had better results than radiologists (91% vs. 59%). The integration of AI into clinical practice is raising new challenges while offering considerable opportunities. It is helping to improve the accuracy of diagnoses, optimize administrative tasks, and personalize treatment plans. Moreover, AI allows healthcare staff to spend more time with patients, enhancing the quality of care and the human relationship (59). For example, the authors showed that a BM segmentation system based on DL can be optimally applied to improve the efficiency of BM delineation in clinical practice (60). Another study has developed DL models for the purpose of proposing an alternative solution for patient-specific quality assurance that would make treatment machines more available to patients and thus enable more patients to be treated (61).

In summary, while significant progress has been made in early characterization and prediction of treatment efficacy in GBM and BM using imaging biomarkers, radiomics, and AI, further research is needed to address current limitations and explore new avenues. Integrating functional imaging biomarkers, updating AI models to reflect recent architecture, and investigating new treatment modalities are key areas for future development.
